# A Valuable Contribution toward Adopting Systematic Review in Environmental Health

**DOI:** 10.1289/ehp.1307701

**Published:** 2014-01-01

**Authors:** Jennifer McPartland, Juleen Lam, Colleen Lanier-Christensen

**Affiliations:** 1 Environmental Defense Fund, Washington, DC, USA; 2 Department of Health, Policy and Management, Johns Hopkins Bloomberg School of Public Health, Baltimore, Maryland, USA; 3 Department of Sociomedical Sciences, Columbia University Mailman School of Public Health, New York, New York, USA

Scientific and regulatory disagreements and debates routinely arise during the determination of which specific environmental agents are of concern and at what exposure levels. Such debates are common during the development of human health assessments by the U.S. Environmental Protection Agency (EPA) Integrated Risk Information System (IRIS). Indeed, IRIS has been the subject of several congressional hearings and National Academy reviews (U.S. EPA 2012; U.S. House of Representatives Committee on Science, Space, and Technology, Subcommittee on Oversight 2011) precisely because it is in the contentious position of assessing chemical hazard. Recently, IRIS has put forth significant effort to enhance and increase the efficiency of its reviews, which includes refining the process by which it selects, evaluates, and integrates scientific evidence (U.S. EPA 2013)—all central elements of systematic review that require transparent and objective criteria or protocols.

In “Instruments for Assessing Risk of Bias and Other Methodological Criteria of Published Animal Studies: A Systematic Review,” Krauth et al. (2013) provided a much needed initial overview of various instruments proposed to evaluate animal study quality. In our view, four particularly valuable contributions of the paper will be useful to IRIS and others seeking to adopt systematic review approaches for environmental health. First, the authors systematically identified instruments currently available to assess animal study quality. This is the first review of its kind and is invaluable for the further development of such instruments. Second, the authors highlighted the considerable variability found between instruments with regard to origin, number, and type (e.g., risk of bias, reporting) of evaluation criteria. Clearly, application of these different instruments will lead to different conclusions given this degree of variability. The authors’ discussion of differences between criteria related to risk of bias, reporting, and imprecision provides important insight on how different criteria can affect study estimates and, consequently, how each should be considered in the evaluation of evidence. Third, the authors described serious uncertainties regarding the performance of these instruments that must be considered in their application. Two of the findings by Krauth et al. (2013) were particularly striking: First, of the 30 instruments evaluated, only 1 had been tested for validity; and second, only 6 contained at least one criterion empirically supported to systematically bias effect sizes in animal studies. The possibility that these instruments would be used to assert definitive conclusions of study quality is especially worrisome given the current lack of empirical support for their design. Last, the authors pointed to specific criteria that need additional research to determine whether they introduce systematic bias, such as timing of exposure, sex, and funding sources and financial ties of investigators. Building the evidence base around these criteria would improve the development of future instruments intended to evaluate experimental animal studies.

In the clinical field, objective and transparent evidence-based systematic review methods have been used for several decades. These methods have been empirically tested and refined over time for evaluating scientific evidence to assess the effectiveness and potential risks of medical interventions (Guyatt et al. 2011; Higgins and Green 2011). Such standardized review methods are desperately needed for the field of environmental health in order to assess potential human health or environmental impacts of chemical exposures.

In their paper, Krauth et al. (2013) provided a critical overview of instruments available for assessing animal study quality and they indicated where additional research is needed to assess and improve these instruments. We believe this review is extremely valuable to entities such as IRIS and the National Toxicology Program Office of Health Assessment and Translation in their current efforts to establish formal systematic review processes when making authoritative determinations of chemical hazard. In particular, it is evident that the differences and uncertainties identified between available instruments assessed by Krauth et al. (2013) must be addressed if public health protective decisions are to be ensured. We strongly believe that the findings of Krauth et al. make apparent the need for a similar review to be conducted on study evaluation instruments and systematic review approaches currently being developed specifically for application to environmental health.
